# BioGlue^®^ Induced Mass Formation Aggravating Spinal Canal Invasion After Intradural Tumor Surgery

**DOI:** 10.3390/jcm14134540

**Published:** 2025-06-26

**Authors:** Sun Woo Jang, Sang Hyub Lee, Hong Kyung Shin, Sang Ryong Jeon, Danbi Park, Chongman Kim, Jin Hoon Park

**Affiliations:** 1 Department of Neurological Surgery, Gangneung Asan Hospital, University of Ulsan College of Medicine, Gangneung 25440, Republic of Korea; sunwoo0118@naver.com; 2 Department of Neurosurgery, Spine Center, The Leon Wiltse Memorial Hospital, Suwon 16499, Republic of Korea; shlee.kns@gmail.com; 3 Department of Neurological Surgery, Asan Medical Center, University of Ulsan College of Medicine, Seoul 05505, Republic of Korea; mxshin@gmail.com (H.K.S.); srjeon190@gmail.com (S.R.J.); danbi08@kakao.com (D.P.); 4 College of Nursing, Korea University, Seoul 02841, Republic of Korea; 5 Department of Industrial and Management Engineering, Myongji University, Seoul 03674, Republic of Korea; chongman@mju.ac.kr

**Keywords:** cerebrospinal fluid leak, intradural spinal cord tumor, spinal canal invasion, BioGlue^®^

## Abstract

**Background/Objectives**: The aim of this study was to evaluate whether the use of BioGlue^®^ increases the risk of postoperative mass formation and subsequent spinal canal invasion after intradural spinal tumor surgery. **Methods**: After retrospectively reviewing patients who underwent intradural tumor surgery from 2018 to 2023, we evaluated mass formation as detected in postoperative MRI according to the Epidural Spinal Cord Compression (ESCC) grade. Patients were divided into two groups based on the use of BioGlue^®^, and we analyzed MRI postoperatively to compare the differences in ESCC grades and the incidence of symptomatic spinal canal invasion between the two groups. Additionally, we performed a logistic regression analysis to identify risk factors associated with mass formation and to explore their relationship with BioGlue^®^. **Results**: This study included a total of 153 patients, 87 in the BioGlue^®^ and 66 in the non-BioGlue^®^ groups. In the BioGlue^®^ group, 18 patients had ESCC grade 2, and 11 had grade 3. Conversely, in the non-BioGlue^®^ group, only 8 patients had ESCC grade 2, and none had grade 3 (*p* = 0.001). Among the cases of symptomatic spinal canal invasion, all five cases were identified in the BioGlue^®^ group (*p* = 0.001). Both univariate and multivariate analyses showed that BioGlue^®^ was a significant risk factor for spinal canal invasion (univariate: OR = 3.931, *p* = 0.005, multivariate: OR = 3.812, *p* = 0.003). **Conclusions**: Our findings indicated that BioGlue^®^ was a significant risk factor for mass formation aggravating spinal canal invasion after intradural tumor surgery.

## 1. Introduction

BioGlue^®^ (CryoLife, Atlanta, GA, USA) is a surgical sealant consisting of bovine serum albumin and glutaraldehyde (BSAG) that polymerizes within 20 to 30 s when mixed. A common adjunct to achieve hemostasis during the surgical repair of large vessels such as the aorta, femoral, and carotid arteries [1,2], BioGlue^®^ is also frequently used in off-label applications in neurosurgery to prevent cerebrospinal fluid (CSF) leakage. This has been supported by several reports discussing its effectiveness in CSF leakage repair [3,4,5,6]. However, the product has yet to receive official, full approval for such use [3,7,8], mainly due to concerns about mass effects, neurotoxicity, and infection [9,10,11]. Reports in the Manufacturer and User Facility Device Experience (MAUDE) databases have routinely documented mass effects associated with BioGlue^®^ used in dural repairs. While a few case reports exist on the side effects of BioGlue^®^ as a dural sealant [5,12,13], a more comprehensive, systematic study is yet to be published. Moreover, in South Korea, BioGlue^®^ is covered by insurance to prevent CSF leakage in neurosurgical procedures. Thus, we aimed to assess the potential risks associated with the use of BioGlue^®^, particularly its relationship with spinal canal invasion and neurological deterioration. We compared radiologic and surgical outcomes between BioGlue^®^ and non-BioGlue^®^ groups to identify its adverse effects in spine surgery.

## 2. Materials and Methods

This study was approved by the Institutional Review Board (IRB, No. 2023-1613), ensuring compliance with ethical standards. Given the retrospective nature of the study, the IRB waived the requirement for informed consent.

### 2.1. Study Design and Participants

We retrospectively reviewed 250 patients who received intradural spinal cord tumor removal surgery from March 2018 to January 2023 and selected 153 after excluding those with any of the following: (1) epidural spinal cord tumor surgery without durotomy, (2) revision surgery, (3) procedure other than laminoplastic laminotomy, and (4) no postoperative MRI or insufficient medical record.

### 2.2. Surgical Procedures

**Figure 1:**
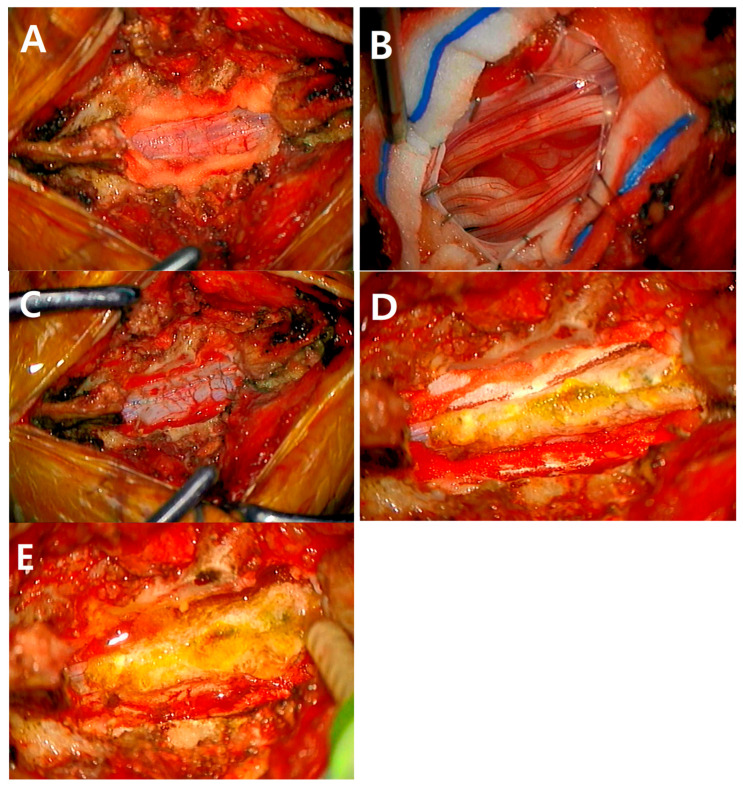
Surgical procedure for spinal cord tumor surgery. Surgical laminectomy or laminoplastic laminotomy was performed at the spinal cord tumor (**A**), and midline durotomy was performed to expose the spinal cord tumor (**B**). After spinal cord tumor resection, dura was closed using Prolene 5-0 and 6-0 sutures (**C**), and adjunctive materials such as a collagen patch (TachoComb^®^; Baxter, Inc., IL, USA) or non-soluble glue (BioGlue^®^) were applied selectively (**D**). A 1 mm-thin layer of BioGlue^®^ was applied to the epidural space as recommended in a previous study to avoid any post-operative mass effect [14] (**E**). Then, laminoplasty was performed depending on the surgical plan, followed by layer-by-layer muscle and skin closure using Vicryl and nylon.

We performed laminoplastic laminotomy at the surgical level on the spinal cord tumor (Figure 1A) and midline durotomy to expose the spinal cord tumor (Figure 1B). After removing the spinal cord tumor, we closed the dura using Prolene 5-0 and 6-0 sutures (Figure 1C), and the lateral side dura defect from the inside in the same manner after removing a dumbbell-shaped schwannoma. Moreover, we selectively applied adjunctive materials such as a collagen patch (TachoComb^®^; Baxter, Inc., Deerfield, IL, USA) or non-soluble glue (BioGlue^®^) based on the surgeon’s decision. In cases, we selectively used either BioGlue^®^ or TachoComb^®^. However, in instances of durotomy involving more than two levels or a weak dura, we employed both products (Figure 1D). To avoid postoperative mass effects, we applied a 1 mm-thin layer of BioGlue^®^ to the epidural space as recommended in a previous study [14] (Figure 1E). Finally, we performed the Valsalva maneuver to screen for unresolved CSF leakage and laminoplasty, followed by layer-by-layer muscle and skin closure using Vicryl and nylon.

### 2.3. Postoperative Follow-Up

In the postoperative state, patients were encouraged to start ambulation on the day after surgery. A follow-up MRI was performed within one week postoperatively to screen for any abnormal findings, such as residual tumor or CSF leakage. If nothing specific was detected on the postoperative MRI and signs of infection or wound dehiscence were not found, the patients were discharged and asked to revisit the outpatient clinic in a month. After confirming the pathology, the follow-up plan for the patients was established. If confirmed as a benign tumor, a follow-up MRI was planned three years after surgery. If diagnosed as a malignant tumor, short-term follow-up with adjuvant therapy such as radiotherapy or chemotherapy was scheduled. During the follow-up period, revision surgery was performed if postoperative complications such as CSF leakage, wound infection, or neurologic deterioration—such as motor weakness due to a mass formation invading the spinal canal found in follow-up MRI—occurred.

### 2.4. Radiological Analysis

By examining the postoperative MRI of the 153 enrolled patients, we revealed that spinal canal invading lesions appeared as a low signal on T2 images. While the epidural lesions could initially be considered as hematomas in the postoperative state, hematomas were isodense on T2 MR images, gradually becoming hypointense and then hyperintense after one week in the hyperacute stage [15,16]. On the other hand, epidural compressing lesions found as hypointense on T2 MR images in this study remained in a follow-up MRI conducted a few months after surgery, suggesting they were not simple hematomas. Additionally, BioGlue^®^ is known to manifest a low signal in T2 MR images [17,18]. Hence, it was assumed that a mass displaying low signal intensity in a T2 MR image was directly correlated with the effects of BioGlue^®^.

The postoperative MRI results were classified based on the Epidural Spinal Cord Compression (ESCC) grading system, originally designed to differentiate the extent of cord compression in metastatic spine tumors to guide surgical intervention [19]: grade 1 with epidural extension without cord compression; grade 2 with spinal cord compression and visible CSF around the spinal cord; and grade 3 with spinal cord compression and no visible CSF near the spinal cord. The patients were categorized into the BioGlue^®^ and the non-BioGlue^®^ groups, depending on the post-dural suture use of BioGlue^®^, to compare the incidences of ESCC grades.

### 2.5. Postoperative Complication Analysis

Postoperative complications requiring revision surgery were collected and classified into the BioGlue^®^ and non-BioGlue^®^ groups to analyze the relationship between the incidence of revision surgery and the use of BioGlue^®^.

### 2.6. Analysis of ESCC Grades 2 and 3 Risk Factors

To diversify our perspectives, we also analyzed numerous risk factors for spinal canal invasion with ESCC grades 2 and 3, including age, sex, type of operation, surgical location, surgical level, and the use of BioGlue^®^ or Tachocomb^®^, as well as the relationship between these factors and ESCC grades 2 and 3.

### 2.7. Statistical Analysis

Simple *t*-tests to compare the BioGlue^®^ and non-BioGlue^®^ groups were conducted using SPSS version 20.0 (IBM, Chicago, IL, USA), with statistical significance set at *p* < 0.05. Logistic regression was conducted to generate estimates of odds ratios (OR) and 95% confidence intervals (CIs) to examine the relationship between the investigated risk factors and ESCC grades 2 and 3.

## 3. Results

### 3.1. Demographics

Table 1 presents the demographic data of the enrolled patients categorized into three groups stratified by ESCC grade. The ESCC grade 1 group had 116 patients, with 51 men (43.9%) and a mean age of 52.8 ± 16.4. The ESCC grade 2 group had 26 patients, with six men (23.1%) and a mean age of 53.2 ± 16.6. The ESCC grade 3 group had 11 patients, including five men (45.4%) and a mean age of 52.9 ± 16.4. Among the 116 ESCC grade 1 patients, 25 (21.5%) were diagnosed with meningioma, 63 (54.4%) with schwannoma, and 13 (11.2%) with ependymoma. Among the 26 ESCC grade 2 patients, 8 (30.7%) were diagnosed with meningioma, 10 (38.4%) with schwannoma, and 6 (23.1%) with ependymoma. Among the 11 ESCC grade 3 patients, 3 (27.3%) were diagnosed with meningioma, 4 (36.3%) with schwannoma, and none (0%) with ependymoma. The three groups showed no statistically significant difference in tumor type (*p* = 0.272).

Next, 29 (25%) patients had a cervical level, 49 (42.2%) a thoracic level, and 38 (32.8%) a lumbosacral level spinal cord tumor in the ESCC grade 1 group; 13 (50%) patients had a cervical level, 10 (38.4%) a thoracic level, and 3 (11.6%) a lumbosacral level spinal cord tumor in the ESCC grade 2 group; and 5 (45.4%) patients had a cervical level, 4 (36.3%) a thoracic level, and 2 (18.3%) a lumbosacral level spinal cord tumor in the ESCC grade 3 group, proving no statistically significant difference between the three groups (*p* = 0.141).

A total of 26 (22.4%) patients had one level surgery, 71 (61.2%) two levels, and 19 (16.4%) three levels or more in the ESCC grade 1 group; 1 (3.9%) patient had one level surgery, 13 (50%) two levels, and 12 (46.1%) had three levels or more in the ESCC grade 2 group; and 3 (27.3%) patients had one level surgery, 5 (45.4%) two levels, and 3 (27.3%) had three levels or more in the ESCC grade 3 group, also confirming no statistically significant difference between the three groups (*p* = 0.761).

Finally, 107 (92.2%) patients in ESCC grade 1 groups, 24 (92.3%) in grade 2 groups, and 9 (91.8%) in grade 3 groups used TachoComb^®^, which revealed no statistically significant difference (*p* = 0.240). However, 58 (50%) patients in the ESCC grade 1 groups, 18 (69.2%) in ESCC grade 2 groups, and 11 (100%) in ESCC grade 3 groups used Bioglue^®^, which revealed a statistically significant difference between the three groups (*p* = 0.001).

### 3.2. ESCC Grade Analysis

A comparison of the postoperative MRIs of the BioGlue^®^ and non-BioGlue^®^ groups using the ESCC grade scale demonstrated that 58 patients (66.7%) in the BioGlue^®^ group had grade 1, 18 (20.7%) had grade 2, and 11 (12.6%) had grade 3, while 58 patients (87.9%) in the non-BioGlue^®^ group had grade 1, 8 (12.1%) had grade 2, and no patient had grade 3. This confirmed a statistically notable difference between the two groups (*p* value = 0.001) (Table 2).

### 3.3. Review of Revision Cases

**Figure 2:**
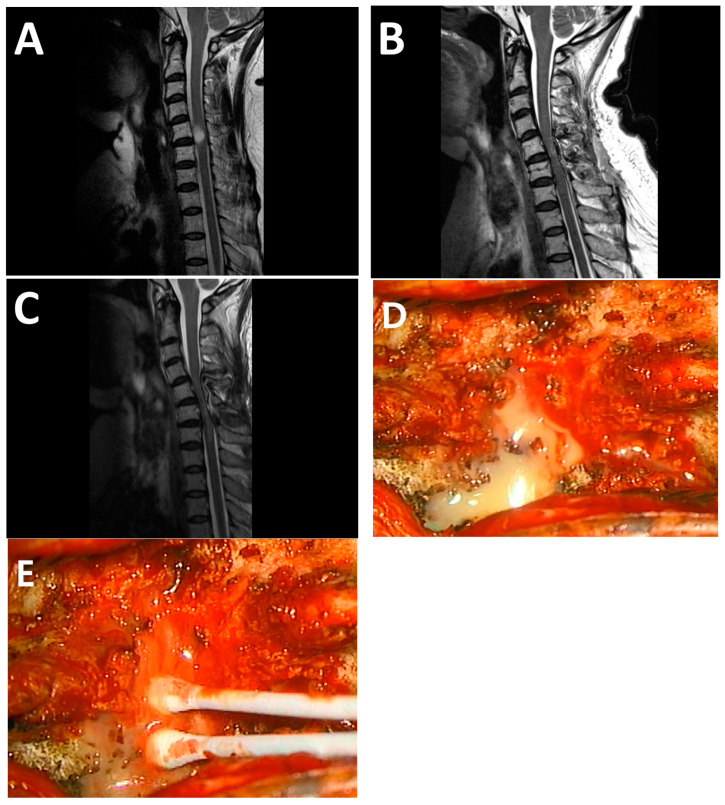
Representative cases of symptomatic cord compression and revision surgery. A 50-year-old female patient was diagnosed with an intradural spinal cord tumor at C5-6 level (**A**) and underwent laminoplastic laminotomy at C5-6 with resection of the intradural spinal cord tumor. Postoperative MR revealed no residual tumor (**B**), and pathology confirmed the tumor as a neurofibroma. Two months postoperatively, the patient experienced weakness in all four extremities, and a follow-up MRI confirmed a severe cord compressing lesion (**C**), leading to her readmission for further evaluation. Initially, a laboratory study was conducted under the suspicion of a postoperative abscess, but it found no clinical signs or lab abnormalities suggesting an infection. Ultimately, diagnostic revisional surgery was performed. During the revision surgeries, a mass compressing the spinal cord was identified, accompanied by yellowish fluid and moderate to severe spinal cord adhesions (**D**). However, culture studies of the yellowish pus in the postoperative state did not detect any specific bacteria or signs of infection (**E**), discontinuing the empirical antibiotics used initially.

Out of the 153 enrolled patients, a total of seven cases required revision surgery due to postoperative complications at the surgical site (Table 3). Among them, five cases were correlated with delayed mass formation aggravating spinal canal invasion. A 50-year-old female patient was diagnosed with an intradural spinal cord tumor at the C5-6 level (Figure 2A) and underwent laminoplastic laminotomy at C5-6 with resection of the intradural spinal cord tumor. Postoperative MR revealed no residual tumor (Figure 2B), and pathology confirmed the tumor as a neurofibroma. Two months postoperatively, the patient experienced weakness in all four extremities, and a follow-up MRI confirmed a severe cord compressing lesion (Figure 2C), leading to her readmission for further evaluation. Initially, a laboratory study was conducted under the suspicion of a postoperative abscess, but it found no clinical signs or lab abnormalities suggesting an infection; ultimately, a diagnostic revisional surgery was performed. During the revision surgeries, a mass compressing the spinal cord was identified, accompanied by a yellowish fluid inside with moderate to severe spinal cord adhesions (Figure 2D). However, culture studies of the yellowish fluid in the postoperative state did not detect any specific bacteria or signs of infection (Figure 2E). Furthermore, no white blood cells or epithelial cells were detected in the sample. In four other patients, postoperative neurological deterioration was observed, with mass formation invading into the spinal canal identified in their follow-up MRI, ranging from POD 1 to 8 months, prompting revision surgeries. All five cases belonged to the BioGlue^®^ group.

Additionally, one patient in the BioGlue^®^ group had a follow-up MRI confirming operative site bulging and CSF leakage. Another case involved a postoperative pulmonary embolism; non-vitamin K antagonist oral anticoagulants (NOAC) were started on day three after surgery. Neurological deterioration and hematoma were identified on follow-up MRI, necessitating revision surgery.

### 3.4. Analysis of Revision Cases

In the seven revision cases, five were confirmed mass formations invading the spinal canal at least one month after surgery, with hematoma ruled out. These were categorized as the symptomatic spinal canal invasion group. They were further divided into BioGlue^®^ and non-BioGlue^®^ groups and underwent a simple t-test, which showed a statistically significant difference (*p* = 0.001) between the two groups. However, no statistically notable difference was found (*p* = 0.080) when the CSF leakage case was compared between the two groups (Table 2). Importantly, all seven revision surgeries occurred exclusively in the BioGlue^®^ group. While only five of these were directly attributable to BioGlue^®^-associated mass formation, and the hematoma case was likely unrelated to BioGlue^®^ use, the exclusive occurrence of all revision cases within the BioGlue^®^ cohort may still warrant caution and further investigation regarding its clinical safety profile in intradural tumor surgery.

### 3.5. Logistic Regression Analysis for Risk Factors of ESCC Grades 2 and 3

We divided the 153 enrolled patients into ESCC grade 1, 2, and 3 groups to analyze the relationship between BioGlue^®^ and ESCC grades from a different perspective. The univariate logistic analysis for ESCC grades 2 and 3 revealed that the use of BioGlue^®^ had an OR of 3.931 with a *p*-value of 0.005, while Tachocomb^®^ was not statistically significant with a *p*-value of 0.685. Furthermore, the cervical location was associated with an OR of 3.524 and a *p*-value of 0.047. A multivariate analysis was conducted on the categories with significant *p*-values and ORs in the univariate analysis, demonstrating an OR of 3.812 with a *p*-value = 0.003 for BioGlue^®^. On the other hand, cervical and thoracic locations had ORs less than 1, which was contrary to that observed in the univariate analysis: cervical location showed an OR of 0.196 with a *p*-value of 0.005, and thoracic location had an OR of 0.394 with a *p*-value of 0.107 (Table 4).

## 4. Discussion

This study identified a significant association between the use of BioGlue^®^ and increased rates of postoperative mass formation, spinal canal invasion, and higher ESCC grades. Among 153 patients, most ESCC grade 2 or 3 cases were found in the BioGlue^®^ group (Table 2). Notably, all five patients who underwent revision surgery for symptomatic spinal canal invasion belonged to the BioGlue^®^ group (Table 3), and logistic regression analysis further confirmed BioGlue^®^ as an independent predictor of higher ESCC grade (Table 4). These findings suggest that BioGlue^®^ may contribute to a clinically significant mass effect following intradural tumor surgery.

Figure 2 presents a representative case in which a mass lesion persisted up to 9 months postoperatively and required surgical re-exploration. The lesion was clearly distinct from hematoma or tumor recurrence, and its MRI signal characteristics were consistent with previously reported BioGlue^®^-associated granulomas, or “Gluomas” [12]. Although causality cannot be definitively established, the consistency of imaging features, surgical findings, and their concordance with prior literature provides strong circumstantial evidence supporting this association.

Although all surgeries were performed by a single surgeon using a standardized thin-layer application technique in accordance with published guidelines [14], mass formation still occurred in some patients. Intraoperative findings and negative culture results ruled out infectious etiologies in these compressive lesions, suggesting an alternative mechanism. This discrepancy between intended technique and adverse outcomes implies that factors beyond surgical application—such as localized anatomical constraints, pooling of adhesive, or individual tissue responses—may contribute to delayed BioGlue^®^-associated spinal canal invasion.

Widely used in cardiac surgery [1,2], BioGlue^®^ has been extensively adopted in neurosurgery, including both cranial and spinal procedures, despite the manufacturer not providing assurances for its safety in spine surgery [4,6]. Various studies, including those by Miscusi et al., have concluded that BioGlue^®^ in spine surgery is an effective adjunct to immediate dural repair in non-instrumented spinal surgery [3]. Its efficacy and safety were comparable to those of other sealants, potentially decreasing the incidence of associated short- and long-term complications. Yuen et al. reported a case demonstrating the persistence of BioGlue^®^ at the repair site two years after its successful use in aiding dural closure during a lumbar decompressive procedure [5]. However, postoperative cases of gluoma formation causing a mass effect have also been reported. Rasul et al. described two cases where the use of BioGlue^®^ caused a local reaction, forming a granuloma that led to cord compression several years after surgery [12]. Nonetheless, aside from such individual case reports, no case series or systematic research on this topic is available. Considering the factors mentioned above, our study is valuable in that it examines the long-term impacts of using BioGlue^®^ and determines whether its application post-dural closure may cause spinal cord compression and neurological decline.

A previous study recognized BioGlue^®^ as a low signal intensity on T2 MR images, thus differentiable from the CSF or other structures [17]; this aligned with our study findings that lesions caused a mass effect on the follow-up MRIs. However, the MRI findings for ESCC grade 2 or higher in the non-BioGlue^®^ group were not clearly differentiated, posing difficulties in attributing the lesion to BioGlue^®^. While the epidural compressing lesions observed were likely to be hematomas in the non-BioGlue^®^ group, they were not distinctive from BioGlue^®^ in MRI.

Univariate logistic regression analysis also confirmed that surgery at a cervical location was associated with higher-grade ESCC. Such a relationship may be due to the smaller spinal canal in this region compared to the thoracic or lumbar levels, leading to more significant cord compression for the same mass effect [20,21,22,23]. Interestingly, this association was reversed in the multivariate analysis, where cervical location appeared to be a protective factor. While the application technique of BioGlue^®^ was consistent across cases due to the use of a standardized thin-layer method by a single surgeon, we hypothesize that anatomical and procedural differences may account for this finding. Specifically, in cervical cases where laminoplasty was performed, the smaller size of cervical laminae and their reattachment with plates may have resulted in a relatively larger reconstructed spinal canal compared to thoracolumbar laminectomy. This increased canal dimension may have attenuated the mass effect of BioGlue^®^, resulting in a lower ESCC grade.

BioGlue^®^ is known to cause toxic effects and wound infection as a side effect [9,10,11] in addition to the mass effect. The literature has anecdotally reported on the toxic effects of BioGlue^®^, associated with the possible development of aseptic meningitis due to the glutaraldehyde component. However, it is noteworthy that bovine serum albumin (45%) and glutaraldehyde (10%) are mixed in the applicator tip to form the glue, after which the glutaraldehyde no longer exists as a single component. While a prior study has demonstrated the biocompatibility of this sealant [24], it should be applied only extradurally with care. Furthermore, this study did not establish a clear connection between BioGlue^®^ and wound infection but confirmed that all four patients with neurologic deterioration had lesions due to a mass effect, hence not identifying evidence to support the neurotoxicity or wound infection of BioGlue^®^.

This study had several limitations. First, its retrospective, non-randomized design introduces a risk of selection bias. Although all cases involved elective intradural tumor surgeries with intentional durotomies—reducing variability in the extent of dural defect—unmeasured confounding variables such as tumor morphology or dural fragility may have influenced the surgeon’s decision to use BioGlue^®^. For example, dumbbell-shaped tumors requiring lateral dural incisions may have been more prone to CSF leakage and thus more likely to be treated with BioGlue^®^. Although all surgeries were performed by a single experienced surgeon using a standardized thin-layer application technique, the possibility of selection bias affecting outcomes cannot be completely excluded. Furthermore, while a prospective randomized controlled trial could better address these concerns, the potential risk of BioGlue^®^-associated mass effect observed in this study raises ethical concerns regarding the feasibility of such a design. Second, information was lacking on follow-up MRI for patients in the non-BioGlue^®^ group with high-grade ESCC. Among the patients treated without BioGlue^®^ and ESCC grades 2 and 3, only two showed resolutions of the lesion in follow-up MRI, limiting the ability to prove that the immediate post-surgical mass effects in patients not treated with BioGlue^®^ are temporary and resolvable. Third, data on clinical symptoms such as pain or neurological deterioration were missing for patients with high-grade ESCC. Further studies should include changes in patients’ clinical symptoms.

## 5. Conclusions

Our findings indicate that BioGlue^®^ is a significant risk factor for mass formation aggravating spinal canal invasion after intradural spinal cord tumor surgery. Therefore, the use of BioGlue^®^ to address CSF leakage in spine surgery is risky and should be limited.

## Figures and Tables

**Table 1:** Demographics of patients who underwent intradural tumor surgery stratified by ESCC Grade.

	ESCC Grade			*p*-Value
	Grade 1 (n = 116)	Grade 2 (n = 26)	Grade 3 (n = 11)	
Age (yrs)	52.8 ± 16.4	53.2 ± 16.6	52.9 ± 16.4	0.392
Sex				0.923
male	51 (43.9)	6 (23.1)	5 (45.4)	
Pathology				0.272
Meningioma	25 (21.5)	8 (30.7)	3 (27.3)	
Schwannoma	63 (54.4)	10 (38.4)	4 (36.3)	
Neurofibroma	2 (1.7)	0 (0)	1 (9.1)	
Ependymoma	13 (11.2)	6 (23.1)	0 (0)	
Glioma	2 (1.7)	0 (0)	1 (9.1)	
Hemangiobloastoma	3 (2.6)	0 (0)	0 (0)	
Cavernous malformation	2 (1.7)	0 (0)	0 (0)	
Metastasis	3 (2.6)	1 (3.9)	1 (9.1)	
Others	3 (2.6)	1 (3.9)	1 (9.1)	
Tumor location				0.141
Cervical	29 (25)	13 (50)	5 (45.4)	
Thoracic	49 (42.2)	10 (38.4)	4 (36.3)	
Lumbosacral	38 (32.8)	3 (11.6)	2 (18.3)	
Operation level				0.761
1 level	26 (22.4)	1 (3.9)	3 (27.3)	
2 levels	71 (61.2)	13 (50)	5 (45.4)	
≥3 levels	19 (16.4)	12 (46.1)	3 (27.3)	
Use of Bioglue^®^	58 (50)	18 (69.2)	11 (100)	0.001 ***
Use of TachoComb^®^	107 (92.2)	24 (92.3)	9 (81.8)	0.240

ESCC grade: epidural spinal cord compression scale; others included chordoma, neuroenteric cyst, hemangioma, benign cyst, granulation, atypical lymphocyte; *** *p* < 0.001.

**Table 2:** ESCC grade and complications in the BioGlue^®^ and non-BioGlue^®^ groups.

	BioGlue^®^ (n = 87)	Non-BioGlue^®^ (n = 66)	*p*-Value
ESCC Grade			0.001 ***
grade 1	58 (66.7)	58 (87.9)	
grade 2	18 (20.7)	8 (12.1)	
grade 3	11 (12.6)	0 (0.0)	
Symptomatic spinal canal invasion	5 (5.7)	0 (0.0)	0.001 ***
CSF leakage	1 (1.1)	0 (0.0)	0.080

ESCC grade: epidural spinal cord compression scale; CSF: cerebrospinal fluid leakage; *** *p* < 0.001.

**Table 3:** Revision cases due to complications.

No.	Age	Sex	Diagnosis	Operation	BioGlue^®^	Complication
1	56	M	Schwannoma	LP, L1	Yes	POD 1 mo MRI compression lesion
2	30	F	Schwannoma	LP, L5	Yes	POD 4 mo progressing motor weakness, MRI compression lesion
3	38	F	Meningioma	LP, C3-6	Yes	POD 1 mo progressing motor weakness, MRI compression lesion
4	50	F	Neurofibroma	LP, C5-6	Yes	POD 2 mo progressing motor weakness, MRI compression lesion
5	63	F	Meningioma	LP, C5-7	Yes	POD 8 mo progressing motor weakness, MRI compression lesion
6	80	M	Schwannoma	LP, S1	Yes	CSF leakage
7	76	F	Meningioma	LP, S1	Yes	POD 3 d NOAC restart due to pulmonary embolism, and subsequent postoperative epidural hematoma

LP: Laminoplasty; POD: postoperative date; NOAC: non-vitamin K antagonist oral anticoagulants.

**Table 4:** Logistic regression analysis for ESCC Grades 2 and 3.

Variables	Univariable Analysis			Multivariable Analysis		
	OR	95% CI	*p*-Value	OR	95% CI	*p*-Value
Age (Ref.: below 70)						
≥70	2.571	0.907-7.290	0.076			
Sex (Ref.: Female)						
Male	2.060	0.815–5.209	0.127			
Location (Ref.: Lumbosacral)						
Cervical	3.524	1.018–12.197	0.047 *	0.196	0.063–0.610	0.005 *
Thoracic	1.764	0.505–6.164	0.374	0.394	0.127–1.224	0.107
Levels (Ref.: 1 level)						
2 levels	1.225	0.341–4.395	0.756			
≥3 levels	3.176	0.791–12.745	0.103			
Use of BioGlue^®^	3.931	1.514–10.212	0.005 **	3.812	1.570–9.258	0.003 **
Use of TachoComb^®^	1.340	0.327–5.499	0.685			

ESCC grade: epidural spinal cord compression scale; OR: odds ratio; CI: confidence intervals; Ref: reference; * *p* < 0.05; ** *p* < 0.005.

## Data Availability

The raw data supporting the conclusions of this article will be made available by the authors on request.
